# Localization of Rock Acoustic Emission Sources Based on a Spaced Sensors System Consisting of Two Combined Receivers and a Hydrophone

**DOI:** 10.3390/s25041197

**Published:** 2025-02-15

**Authors:** Yuri Marapulets, Albert Shcherbina, Alexandra Solodchuk, Mikhail Mishchenko

**Affiliations:** Laboratory of Acoustic Research, Institute of Cosmophysical Research and Radio Wave Propagation FEB RAS Kamchatka Region, Mirnaya Str. 7, Elizovskiy District, 684034 Paratunka, Russia; albert_pkam@mail.ru (A.S.); aleksandra@ikir.ru (A.S.); micle@ikir.ru (M.M.)

**Keywords:** acoustic emission of rocks, hydrophone, combined hydroacoustic receiver, location of AE sources, 43.58.Gn, 43.58.+z, 43.40.Le

## Abstract

The paper considers the results of experiments on localization of the sources of geoacoustic radiation generated in near-surface sedimentary rocks. Geoacoustic signals from sources were recorded by a spaced sensor system consisting of two combined receivers and a hydrophone. The system was installed near the bottom of a natural water body (Mikizha lake) in Kamchatka. Radiation sources were located by two methods, a triangulation survey and estimation of the signal arrival time difference from spaced receivers. Coordinates for more than 40 sources were measured, and their space distribution was mapped. As the result of the experiment, it was stated that geoacoustic radiation sources are located in bottom rocks at the depths up to 2.20 ± 0.25 m at the distances of up to 8 ± 0.25 m. Localization of geoacoustic radiation sources is topical for projecting a new alarm system for the notification on the possibility of strong earthquake occurrence. The results of the analysis of the frequency of rock AE source generation and accurate estimation of their location will be the basis of this system.

## 1. Introduction

An acoustic emission (AE) is an elastic wave radiation occurring as the result of rearrangement of inner structure in solid environments. Characteristics of this radiation are directly associated with deformation process features. Thus, the passive acoustic emission method became widespread both in non-destructive testing in industry [1,2,3,4] and in geophysics in the investigations of geoacoustic oscillation sources in a wide range of their scale, from cavities in rock samples to earthquake sources [5,6,7,8,9].

In 1990s, there was a series of publications that confirmed the efficiency of application of acoustic emission in the frequency range from hundreds to kilohertz for the tasks of earthquake precursor investigation. It was shown in paper [10] that 16 h before the catastrophic Spitak earthquake with the magnitude M=7.1, occurred in Armenia in 1988, an acoustic emission anomaly lasting for several hours was recorded in the range of 800–1200 Hz. The distance from the observation site to the epicenter was 80 km. The paper [11] shows the results of AE investigations, which were carried out in the main tunnel of Matsushiro seimological observatory. It was shown that AE anomalies were recorded before the earthquakes in Japan during the period from 1996 to 1998. Papers [12,13] show the results of the analysis of the information from deepwater bottom stations with all-round view hydrophones installed in the region of Kamchatka, Kuril islands, and the seas of Okhotsk and Japan. It was shown that such systems record earthquake acoustic signals and anomalous precursor signals. We should note that the results of these works showed for the first time that geoacoustic signals, including earthquake and precursor signals, propagate well during the transition from a solid medium into a fluid. Thus, acoustic emission signals can be effectively recorded by the sensors installed near the bottom in water medium.

As the result of long-term observations of acoustic emission on Kamchatka peninsula, which is part of the Pacific Ring of Fire and is one of the most seismically active regions of the globe, high-frequency acoustic-emission effect was discovered for the first time [14]. This effect is the growth of geoacoustic radiation intensity in the frequency range from hundreds of Hz to the first tens of kHz under the condition of rock mass deformation velocity increase. This effect manifests the most brightly 1–3 days before earthquakes at the distance of first hundreds of kilometers from their epicenters [15]. AE kilohertz anomalies were also recorded several days before earthquakes in Italy [16,17,18,19]. In particular, paper [16] describes the case of recording of rock AE anomaly at the frequency of 25 kHz two days before the earthquake with the magnitude of 4.6.

Due to significant attenuation, AE signals at the frequencies of hundreds of hertz to kilohertz cannot propagate from epicenters of preparing earthquakes, and are the responses of environment at a recording site on the change of its stress–strain state [14]. In this case, accurate localization of AE sources is necessary for constructing a model of rock AE generation at different stages of seismic activity. This is very important for projecting a system of notification on the possibility of occurrence of a strong earthquake. It is possible to determine the direction to a radiation source using a combined receiver, which joins a three-component vector sensor and an omnidirectional one [20]. However, it is more difficult to determine the distance to a source. Analytical estimate of the distance to a source was presented in the papers [20]. It was shown that acoustic signals observed in the experiments are generated by shear sources located at the distances up to the first tens of meters from a receiver. This estimate should be confirmed by the results of experiments in natural conditions. The paper presents the results of the experiments on localization of rock AE sources. The experiments were carried out at Mikizha lake in Kamchatka.

## 2. Recording System for Rock AE

**Figure 1:**
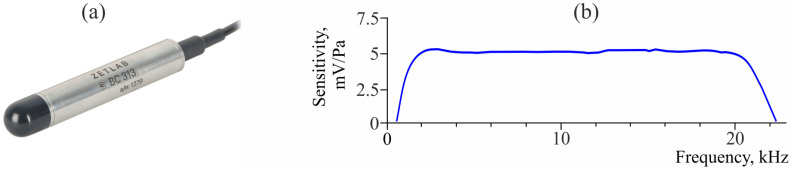
Photo of the Zetlab BC313 hydrophone (**a**) and its channel sensitivity (**b**).

**Figure 2:**
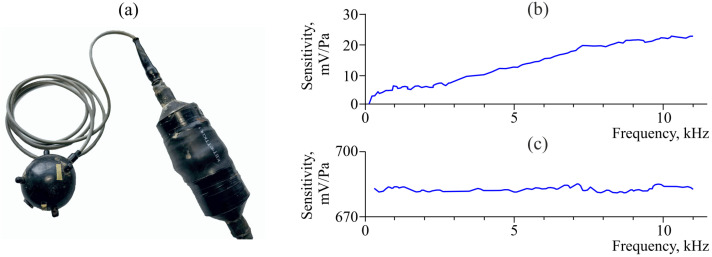
Photo of the combined hydroacoustic receiver with amplifier (**a**). The diameter of the combined receiver is 5 cm. Sensitivity of the pressure gradient channels (**b**) and the pressure channel (**c**).

The peculiarity of the experiments on AE recording in Kamchatka is the application of wide-band hydrophones and vector receivers installed in water near the bottom of natural and artificial water bodies [21]. Application of these receivers made it possible to record acoustic emission within the whole range of sound frequencies (20 Hz–20 kHz). If only AE signal records are required, without solving the task of determination of a sound wave arrival direction, a hydrophone can be used (Figure 1). In order to determine the direction to a radiation source, it is appropriate to use multi-component sensors. Combined hydroacoustic receivers have such characteristics. They combine an omnidirectional receiver of acoustic pressure and a three-component receiver of acoustic pressure gradient (Figure 2). Thus, several parameters of acoustic field are recorded synchronously at one space point. This gives the possibility of recovering the spatial-time distribution of environment particle oscillating speed vector in a wave using vector-phase methods and to determine the direction to a signal source [22].

Figure 1 illustrates Zetlab BC313 hydrophone and sensitivity of its channels, which can be used as an AE signal receiver. The frequency range of this sensor is 20 Hz–20 kHz.

Figure 2 shows the photo of the combined hydroacoustic receiver with external amplifier and receiver channel sensitivity. The receiver consists of a spherical transducer of acoustic pressure and a three-component receiver of pressure gradient, the sensors of which are arranged along mutually orthogonal directions. The receiver case is a hermetic sphere with the diameter of 5 cm, hung up on elastic ropes inside a special frame. The construction is installed at the bottom of the lake and provides constant orientation in space. The operating frequencies of the combined receiver are from 5 Hz to 11 kHz. To normalize the signal level, an outside multi-channel coupling amplifier is used. The receiver records simultaneously the acoustic pressure P(t) and three mutually orthogonal components of its gradient $P_{x}\left(t\right)$, $P_{y}\left(t\right)$, $P_{z}\left(t\right)$. Pressure gradient components are equal to the projections of pressure gradient vector on the corresponding coordinate axes. Applying the vector-phase methods to these four signals, we can determine the vectors of oscillation velocities, shift, and acoustic emission power density [22].

A typical rock AE signal, recorded in water bodies in Kamchatka, consists of a sequence of relaxation pulses of different amplitude and duration with the filling frequency from hundreds of hertz to tens of kilohertz. The repetition frequency during calm periods is units per second and, during anomalies preceding seismic events, it reaches tens or even hundreds per second [14]. The results of experimental investigations in closed inner water bodies [14] and on the ocean shelf [23,24] showed that distortion of pulse signal form, when they propagate at short distances in a waveguide consisting of a water layer and a near-surface soil layer, is not significant. Thus, an investigation of rock geoacoustic signals by a receiver, installed near the bottom of a water body, is quite conceivable. We should note that there are no transverse oscillations in fluids. That makes it possible to detect naturally the longitudinal waves propagating in solid mediums. At the boundary of solid and fluid mediums there is refraction. The refraction coefficient is about 1.2–1.7 during the transition of longitudinal oscillations from sedimentary rocks into water.

**Figure 3:**
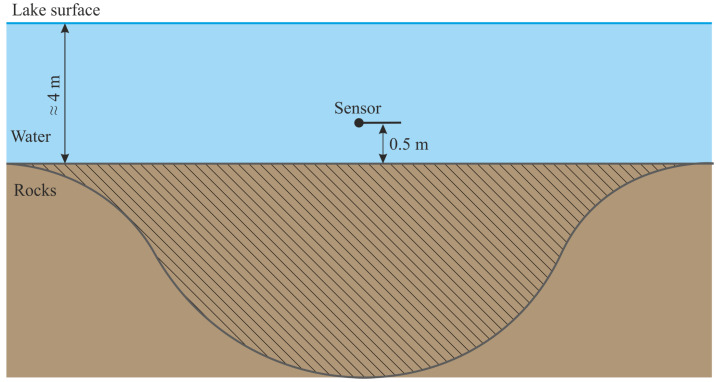
Scheme of hydroacoustic receivers installation. The vertical section through the point of the receiver installation is illustrated. The region from which signal can be received is crosshatched.

In the experiments described in this paper, the sensors were installed near the bottom of Mikizha lake (52.99° N, 158.23° E). The scheme of sensor location is illustrated in Figure 3.

We developed a system for collecting, storing and processing the data recorded by the receivers [21]. Acoustic signals are amplified preliminary and enter the analogue–digital converter. Then, the digitized data are transmitted by wire or wirelessly and stored on a PC hard disk. In case of using the wire connection, the data can be transmitted to the distance up to 200 m. In the case of a wireless connection, the data can be transmitted to the distance up to 1–2 km within the line of sight.

## 3. Determination of the Direction to a Radiation Source by the Combined Receiver

**Figure 4:**
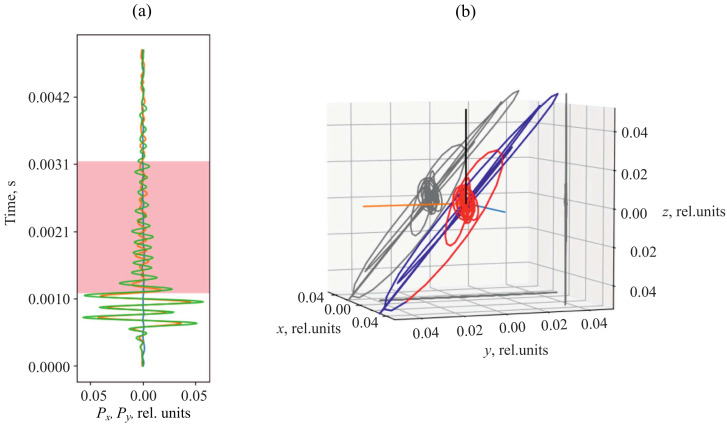
Example of an AE pulse recorded together with the reflected signal. Pulse waveform (**a**). The pink area is a signal fragment formed by direct and reflected waves. The green line $P_{x}$ represents the *x* component of the pressure gradient, and the orange line $P_{y}$ represents the *y* component of the pressure gradient. Hodograph of acoustic pressure gradient (**b**) represented in three-dimensional coordinates with the projections (gray curves) on axial planes. The red line is the reflected wave. In the figure, *x*, *y*, and *z* are coordinate axes.

Determination of the direction to a signal source and the distance to it is described in detail in paper [25]. As an example, we consider the acoustic pulse (Figure 4a) and its three-dimensional representation (Figure 4b). It is clear from Figure 4b that the pulse has elliptic form.

**Figure 5:**
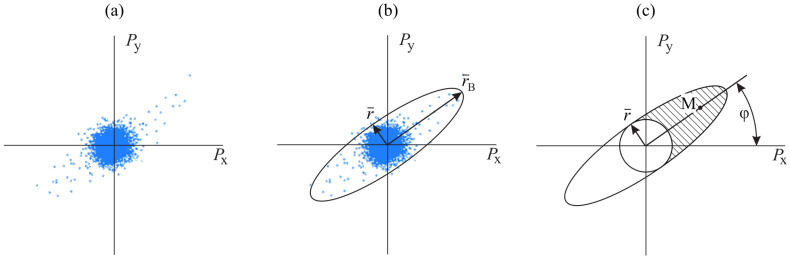
Determination of the direction to a signal source: projections of pulse samples on the xy plane (**a**), mapping of the describing ellipse and determination of noise level (**b**), elimination of count direction ambiguity, estimation of mass center *M* and determination of azimuth on the signal source (**c**). (Reprint from [25]).

In order to determine the direction to a signal source in the horizontal plane xy, the amplitude method is used [22]. In this respect, pulse counts are reflected in the Cartesian coordinate system (Figure 5a). The region where the counts are grouped can be described by an ellipse (Figure 5b). In this case, the semi-major axis of the ellipse ${\bar{r}}_{B}$ corresponds to the sound wave arrival direction. The semi-minor axis $\bar{r}$ almost corresponds to the noise level (Figure 5b).

Due to the symmetry of pressure gradient channel characteristics directivity of the combined receiver, there is uncertainty in the determination of the direction to a signal source. To resolve it, the pressure channel is used. Phase differences of the signals, recorded by pressure channel and pressure gradient channels of the combined receiver, are analyzed. As a result, the majority of the pulse counts with the amplitudes, exceeding the background level, are grouped in one half of the ellipse along its principal semi-axis (Figure 5c). In order to determine the direction to a signal source, we calculate the direction to the mass center *M* of the points from the crosshatched region, describing the ellipse (Figure 5c),

(1) $$\tan\varphi =\frac{M_{y}}{M_{x}},$$

where φ is the azimuth, and $M_{x},M_{y}$ are projections of mass center *M* on the *x* and *y* axes.

To realize this method in space, besides the analysis of pulse projections on the horizontal plane xy, we also consider the projection on two mutually orthogonal vertical planes. We determine the three-dimensional coordinates of mass center *M* and direction to the signal source.

AE direction was estimated for background periods on the days when there were no distinct long acoustic anomalies and during anomalous increases in radiation intensity. It was discovered that acoustic pulse number distribution in space (acoustic activity D(α)) is quite isotropic when there are no anomalies. Anisotropic short (tens of hours) intensive anomalies of rock AEs occur within a 1–3 day interval before earthquakes [20]. Examples of rock AE directivity during background periods and before earthquakes are illustrated in the paper [21].

**Figure 6:**
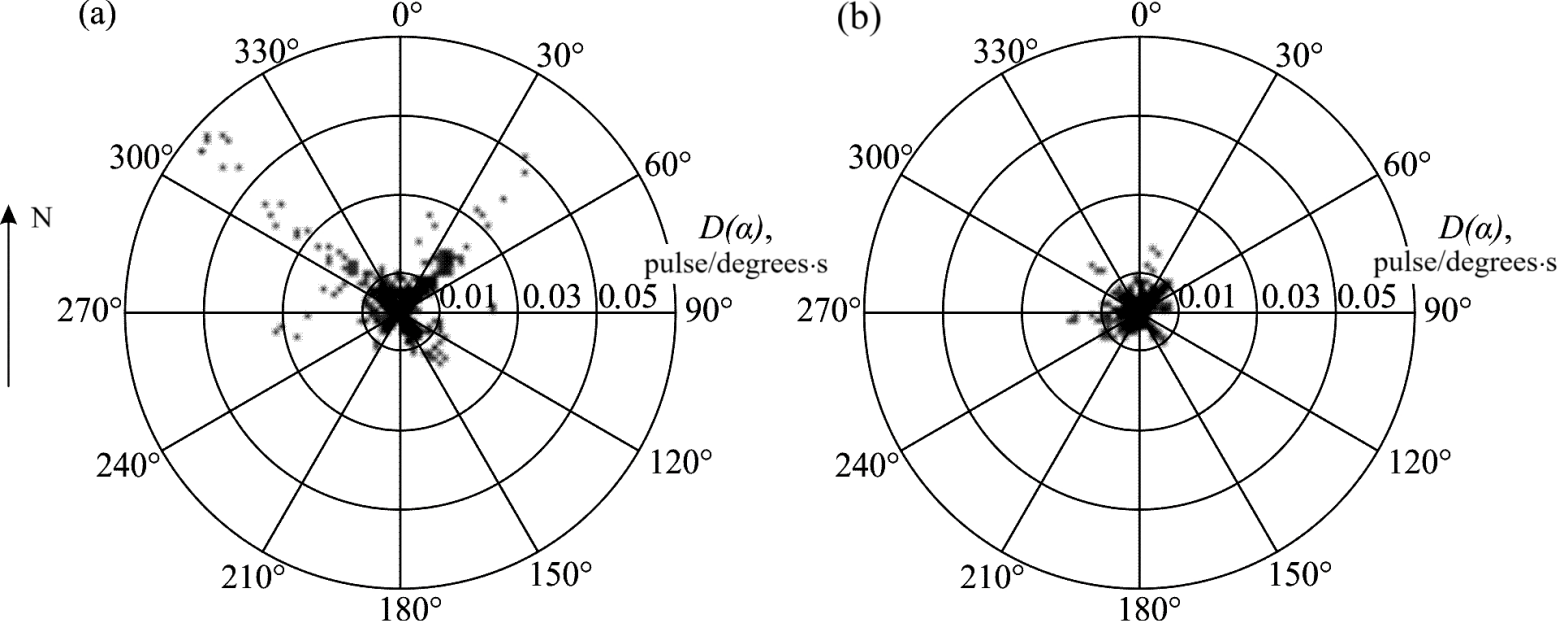
Distribution of acoustic activity D(α) maximums in 2008–2016 during AE anomalies before earthquakes (**a**) and during background periods (**b**) when there were no strong seismic events.

Distribution of acoustic activity D(α) maximums in 111 cases of AE anomalies, recorded within the 3-day interval before earthquakes, was analyzed for the period 2008–2016 [20]. Figure 6a shows generalized results of the analysis of distribution of acoustic activity D(α) maximums for these 111 cases of anomalies. The acoustic activity D(α) maximums are marked on the diagram for each of the anomaly under consideration. It turned out that high acoustic activity was recorded the most frequently from the directions close to 310°–315° and 40°–45°. More rarely, AE pulses were radiated from the directions within the ranges of 130°–165° and 210°–235°. Thus, radiation maximums orientation was determined by two main mutually orthogonal directions to the north-west and north-east. As a comparison, Figure 6b illustrates a diagram of acoustic activity D(α) maximums for 129 background periods of rock acoustic emission with the duration of one day. The periods belong to the same interval of the years. It is clear from Figure 6 that the directions, which were clearly distinguished during earthquake preparation (Figure 6a), differ slightly from other directions during background periods (Figure 6b).

To estimate the distance to a signal source [25], we use the following feature of the combined receiver. The combined receiver, referring to the oscillating-type instrumentation, records superposition of the waves, propagating simultaneously in its vicinity. Thus, we often observe integration of the signal arriving directly from a source and its reflection from water surface (pink area in Figure 4a).

As it is clear from Figure 4b, at the initial phase of the recorded AE pulse, back-and-forth motion of the receiver is observed along the axis directed to the signal source (blue line in Figure 4b). About 0.5 ms later, the hodograph changed (red line in Figure 4b). This is caused by overlapping of the vertical component on the main signal.

**Figure 7:**
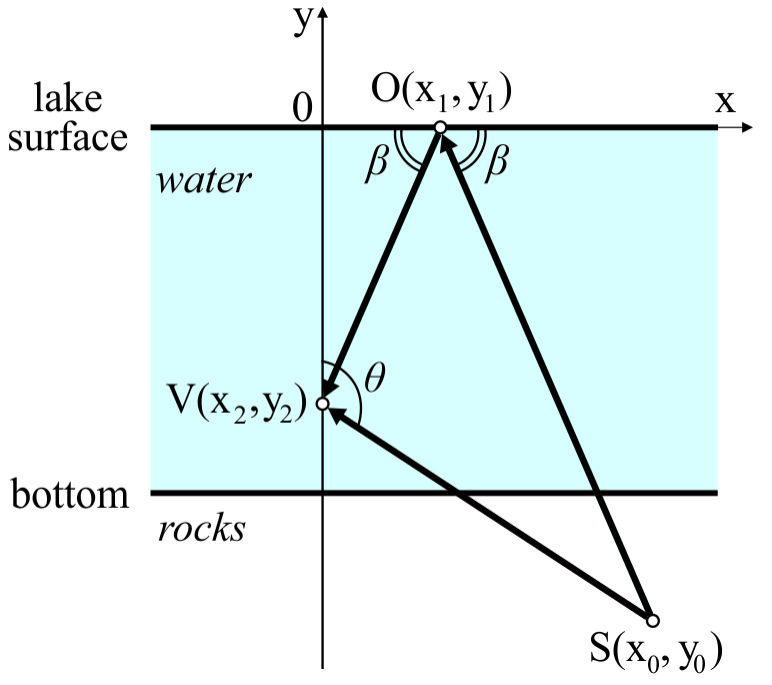
Scheme of direct wave (SV) and reflected wave (OV) propagation. $S(x_{0},y_{0})$ is the source, $O(x_{1},y_{1})$ is the incidence point, and $V(x_{2},y_{2})$ is the receiver. (Reprint from [25]).

We consider the simplest case when the receiver records two waves, the direct one and the one reflected from lake surface (Figure 7). Signal source coordinates are determined from the equation system obtained according to the scheme (Figure 7).

The distance to a source corresponds to the vector SV length,

(2) $$\left|\bar{\mathrm{SV}}\right|=\sqrt{{\left(x_{0}-x_{2}\right)}^{2}+{\left(y_{0}-y_{2}\right)}^{2}}.$$

Thus, the problem is reduced to the finding of signal source coordinates. For that purpose, according to the scheme (Figure 7), we can compose a system of equations.

(3) $$\left\{\begin{array}{c} \begin{array}{cc} \sqrt{{\left(x_{0}-x_{1}\right)}^{2}+{\left(y_{0}-y_{1}\right)}^{2}}+ & \sqrt{{\left(x_{2}-x_{1}\right)}^{2}+{\left(y_{2}-y_{1}\right)}^{2}}-\ldots \\ \; & \ldots -\sqrt{{\left(x_{0}-x_{2}\right)}^{2}+{\left(y_{0}-y_{2}\right)}^{2}}-v\cdot \Delta t=0, \end{array}\\ y_{0}-x_{0}\cdot \tan\left(90^{\circ }-\theta \right)-y_{2}=0,\\ x_{1}=\frac{x_{0}\cdot y_{2}}{y_{0}+y_{2}}, \end{array}\right.$$

where *v* is the sound wave propagation velocity in water, Δt is the delay between the times of recording of the direct and reflected waves, θ is the elevation angle.

The first equation of the system takes into account the time delay Δt. The second one is the equation of line with the directing vector SV. The third one is the sequence of the reflection law. The time delay Δt, sound wave propagation velocity in water *v*, and coordinates of the sensor ($x_{2},y_{2}$) are known to us. We consider the lake surface as zero level. The solution of this equation system is signal source coordinates. Substituting the obtained coordinates into (2), we determine the distance to the source.

The distances to AE sources estimated by the suggested method turned to be within the range of 0.5–5 m that, on the whole, corresponds to the distance estimates presented in the paper [20]. Examples of AE signals with estimated distances to their sources are illustrated in the paper [25]. Such a method of distance estimation may result in quite low accuracy, since it does not take into account sound velocity in rocks and refraction effects during the transition through the interface boundary. To minimize them, an experiment was carried out on the basis of the spaced system consisting of several sensors.

## 4. Localization of Radiation Sources by Spaced System Consisting of Several Sensors

**Figure 8:**
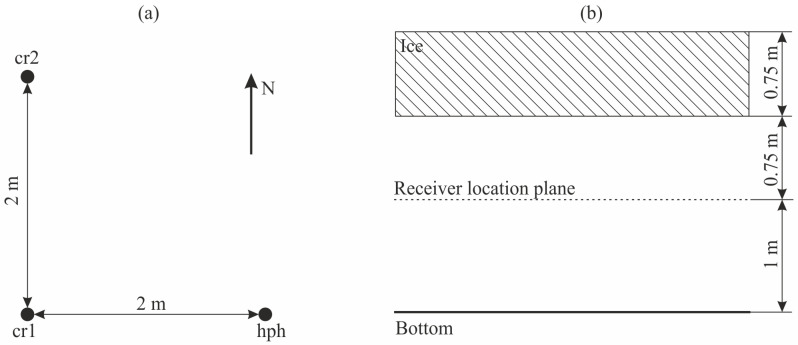
Installation diagram of the spaced receiving acoustic system in the horizontal plane (**a**), in the vertical plane (**b**). cr1 and cr2 are the combined receivers, hph is the hydrophone.

The experiment on localization the AE sources by the spaced system of several sensors was carried out in February 2024 near the bottom of Mikizha lake. At that time, the ice thickness was 0.75 m, the distance from the ice lower edge to the bottom was 1.75 m. To record the signals, a system consisting of two combined receivers (cr1, cr2) and one hydrophone (hph) was installed in the lake (Figure 8). The sensors were horizontally spaced according to three points of a square with the side of 2 m, at the height of 1 m from the bottom.

**Figure 9:**
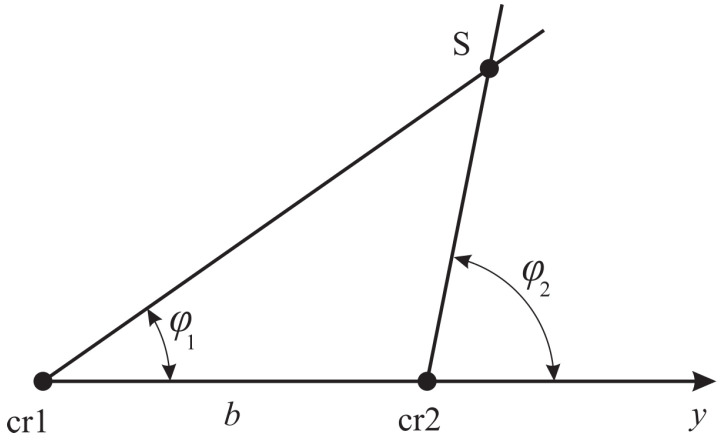
Localization of the *S* signal source by triangulation survey method using two combined receivers cr1 and cr2 spaced at a distance of *b*.

**Figure 10:**
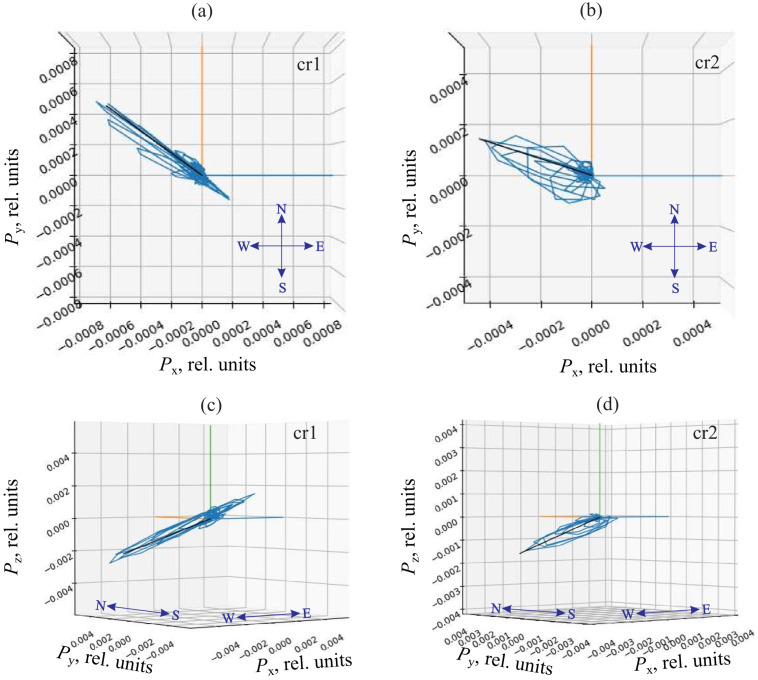
An example of determining the direction to the source of rock pulse AE recorded at 02:01:09.398 on 13 February 2023 by the combined receivers. (**a**,**b**) are the top views; (**c**,**d**) are the side views.

The presence of two combined receivers spaced at a known distance made it possible to determine the location of geoacoustic signal sources using the triangulation survey method. For this purpose, a vector hodograph, collinear to the Umov–Poynting vector, was estimated. It can be used to determine the direction to a signal source [26]. Then directions to signal sources for each receiver are determined. The intersection of the half-lines, corresponding to the obtained directions and built from the receiver centers, determines the signal source location *S* (Figure 9). Figure 10 shows an example of an application of the triangulation survey method to determine geoacoustic signal source location [27].

The system with two combined receivers makes it possible of locate AE sources with high accuracy, if the angles $\varphi_{1}$ and $\varphi_{2}$ (Figure 9) exceed significantly zero values. However, if signal sources are near the straight line, passing through the sensors, then the angles $\varphi_{1}$ and $\varphi_{2}$ has the values close to 0, and location error increases greatly.

To eliminate the problem, it is possible to use the method based on the calculation of the time difference of signal arrivals to the recording components of the system. As was indicated above, the construction of the combined receivers includes omnidirectional sensors of acoustic pressure. Together with the additional sensor of acoustic pressure (hydrophone), the developed system is a system of three receivers spaced at fixed distances. Thus, for more accurate localization of AE sources, the method based on the estimation of the difference of signal arrival times from three spaced receivers was used together with the triangulation survey based on two combined receivers. In the spatial coordinate, the solution of this problem is reduced to the search for the point of intersection of several hyperboloid surfaces of source location, the foci of which correspond to the receivers parameters. To provide accurate measurements, at least four receivers are required, spaced in such a way as to organize three independent spatial bases. Hyperboloid intersection points are determined analytically or by iteration methods of least squares or multi-dimensional optimization.

In our case, there are two pairs of points (cr2–cr1, cr1–hph), allowing us to construct two hyperbolic surfaces of source location with perpendicular axes. The third pair (cr2–hph) is located in the same plane with the first two ones and, as a result, it is poorly suited for the solution of spatial problems. Complex application of the methods of triangulation survey and elimination of the difference of signal time arrival turned to be sufficient for accurate localization of a AE source.

**Figure 11:**
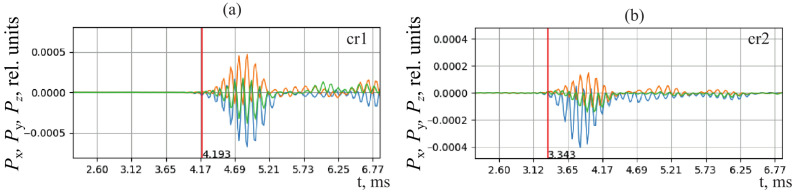
The shape of rock AE pulse recorded at 02:01:09.398 on 13 February 2023 by the combined receivers cr1 (**a**) and cr2 (**b**). Components of the vector collinear to the Umov–Poynting vector: the blue line $P_{x}$ is the direction to the west, the orange $P_{y}$ is the direction to the north, and the green $P_{z}$ is the direction vertically upwards. The red line is a marker of the beginning of the pulse with a time shift relative to the general window. *A* is the amplitude.

In the course of the investigation, it was determined that a signal from a source recorded by different sensors has significant differences in its wave form (Figure 11), which are determined in a greater degree by the nonuniformity of the radiation source directivity diagram [20] and to a lesser degree by the differences in the conditions of signal propagation and recording. This circumstance did not allow us to apply the correlation methods for determining the delay between the signals from different sensors [28]. The signal arrival delay time was estimated either by a pulse front (the time moment when a signal began to exceed the background level) of by the points of its highest value. In both approaches, instantaneous value of recorded acoustic wave amplitude, which depends on oscillation frequency and phase, is used. When using the instantaneous value, an error of determination of pulse arrival time occurs. It is equal to one or several oscillation periods. For the pulse filling frequency of 4 kHz, the error is about 1 m at the distance of 5 m perpendicularly to the combined receiver axis cr1–cr2 and 2.5 m at the same distance in the direction of 45°.

**Figure 12:**
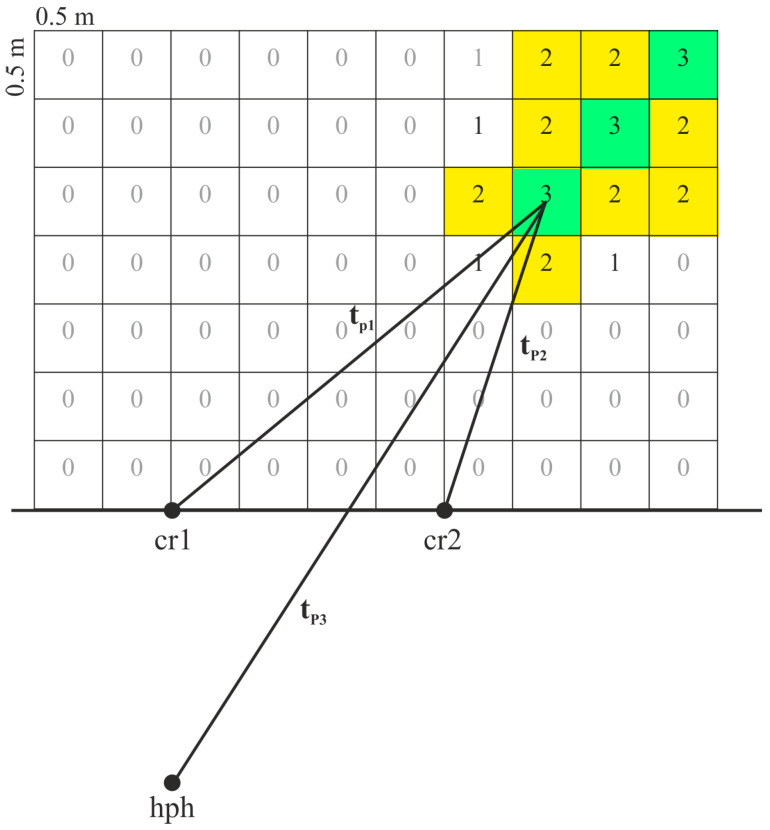
Localization of rock AE sources by empirical implementation of the method based on calculating the difference in signal arrival time. $t_{p1}$, $t_{p2}$, and $t_{p3}$ are estimated times of signal transmission from the investigated element of the controlled volume to the corresponding receiver. Green and yellow colors indicate areas, the direction of which is reliably determined using three pairs of receivers and two pairs of receivers, respectively. The number in the square is the value of *c*.

Taking into consideration the error levels, and the fact that the controlled volume of rock is limited by the lower half-sphere with the diameter of several tens of meters [20,25], it is not reasonable to apply analytical methods to solve the equations of hyperboloids intersection. Taking this into consideration, a simple empirical method for localization of signal sources was used. For this purpose, a volume of bottom rocks in the form of a rectangular parallelepiped with the dimensions of 20 × 20 × 10 m was selected conditionally. The combined receiver cr1 was placed above its center. The volume was divided into equal cubes with the side of 0.5 m, determined by the estimates of source sizes [20] and the determined accuracy of 1–2.5 m (Figure 12). Using a computer program, for each cube, the following function value was estimated sequentially:

(4) $$\;c=c_{12}+c_{23}+c_{13},$$

where

(5) $$\;c_{ij}=\left\{\begin{array}{c} 1,\;\mathrm{if}\;\left|\Delta t_{pij}-\Delta t_{sij}\right|<t_{err}\\ 1,\;\mathrm{if}\;\left|\Delta t_{pij}-\Delta t_{sij}\right|\geq t_{err} \end{array}\right.,$$

where $\Delta t_{pij}$ is the estimated mutual delay between the receivers *i* and *j*, $\Delta t_{sij}$ is the measured mutual delay between the receivers *i* and *j*, and $t_{err}$ is the tolerable error value corresponding to the time of a signal passing the distance equal to 0.25 m (half of the cube side).

The value c=3 for any cube means that all three pairs of the receivers recorded the signal outgoing from this rock volume. In this case, the cube turns green. If c=2, then only two pairs of the receivers determined the direction correctly, the cube color is yellow (Figure 12). If c<2, then such cases have not been considered.

**Figure 13:**
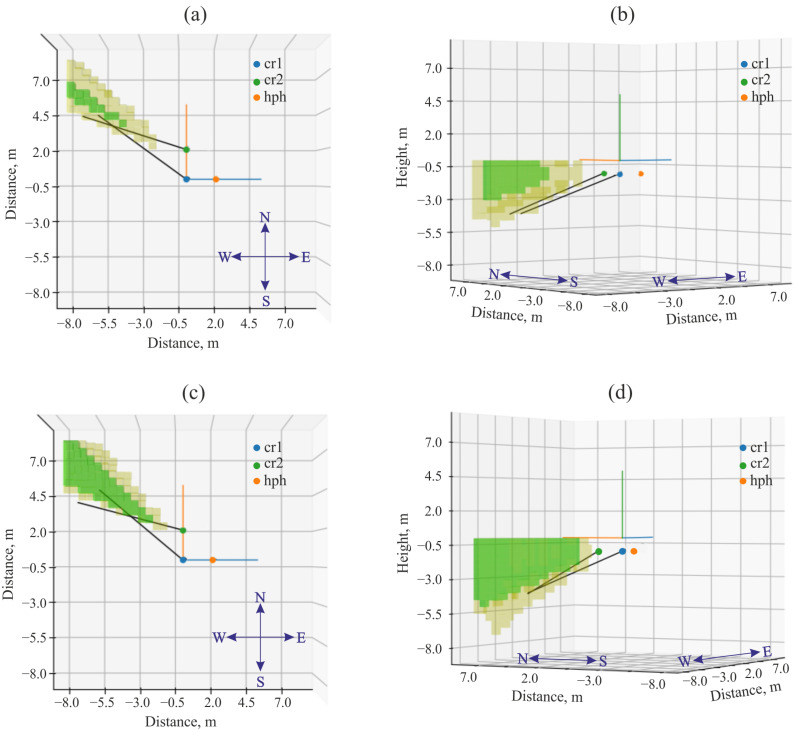
Examples of the localization of rock AE sources by triangulation survey (black rays) and the empirical implementation of the method based on calculation of the difference in signal arrival times (green and yellow areas). The pulse recorded at 02:01:09.398 on 2 February 2023: (**a**,**b**) are the top and side views, respectively. The pulse recorded at 02:01:18.927 on 2 February 2023: (**c**,**d**) are the top and side views, respectively.

Examples of complex application of the triangulation survey method and estimation of the difference of signal arrival times are illustrated in Figure 13. It is clear that the results of AE sources location, obtained by two methods, agree well between each other. Intersection of the half-lines, corresponding to the directions to the signal source during the triangulation survey are in the domain, which is determined as a source location with three or two pairs of pressure receivers. The source location error was less than 0.5 m.

**Figure 14:**
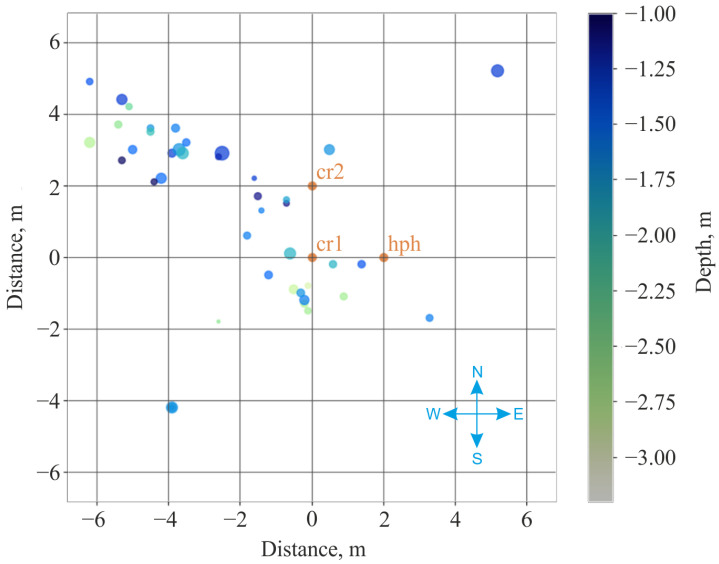
Distribution of signal sources in the horizontal plane.

**Figure 15:**
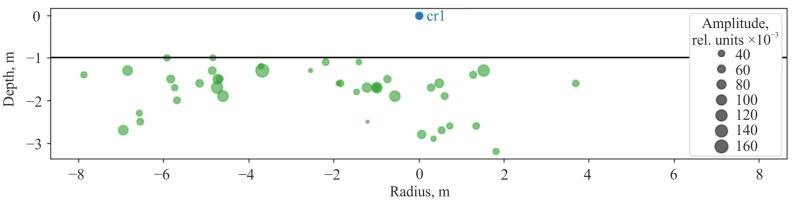
The vertical cross-section of the controlled volume of rocks in azimuth is −55° relative to the combined receiver cr1.

During the experiment time (105 min), 43 pulse signals of rock AE were recorded. Source coordinates were measured for each of them by the methods described above. After that, source location distributions in horizontal plane (Figure 14) and with respect to depth (Figure 15) were constructed. As the result of the analysis of the distributions, it was stated that the sources of the recorded geoacoustic radiation are located in bottom rocks at the depths up to 2.20 ± 0.25 m and at the distances up to 8 ± 0.25 m from the receiver.

## 5. Conclusions

In order to construct a model of AE generation at different stages of seismic activity, exact localization of AE sources is required. It is possible to determine the direction to a radiation source using the combined receiver, which joins a three-component vector sensor and an omnidirectional sensor. However, it is a more complicated task to determine the exact distance to the source. Previous analytical estimates of the distances to radiation sources showed that the distances are up to the first tens of meters from the receiver. To confirm these estimates, experiments were carried out near the bottom of Mikizha Lake in Kamchatka. The experiments were realized in winter conditions when the lake surface was covered with ice.

Initially, the distances to AE sources were estimated on the basis of the difference of arrival of a direct acoustic wave from a source and the wave reflected from the ice on the lake surface. The distances to rock AE sources estimated by the suggested method turned to be within the range of 0.5–5 m. However, this method for distance estimation can result in quite poor accuracy. In order to minimize this, an experiment, based on a spaced system of several sensors, was carried out. Two methods were used jointly to determine rock AE source locations, namely triangulation and estimation of the difference of arrival times of signals from spaced sensors. As a result, it was stated that the sources of recorded geoacoustic radiation were located in bottom rocks at the depths up to 2.20 ± 0.25 m at distances of up to 8 ± 0.25 m. The measurement error was less than 0.5 m.

Thus, the previous analytical estimates of the distances to AE sources were confirmed by the results of the experiments in natural conditions. Results of the analysis of the frequency of rock AE sources generation and accurate estimation of their location will be the basis for a new alarm system for notifications of the possibility of strong earthquake occurrences.

## Data Availability

The data on earthquakes considered in the article are available at Unified Information System of Seismological Data of the Kamchatka Branch of Geophysical Service Russian Academy of Science at http://sdis.emsd.ru/info/earthquakes/catalogue.php (accessed on 15 January 2025).
